# Clinical Impact of Fingolimod-Associated Lymphopenia in Multiple Sclerosis: A Real-World Cohort Study

**DOI:** 10.3390/medicina62091716

**Published:** 2026-09-07

**Authors:** Murat Yeniçeri, Mustafa Can Şenoymak, Süleyman Baş, Hasan Hakan Çoban, Ayşe Gürbüz Yeniçeri, Serkan Demir, Alpaslan Tanoglu

**Affiliations:** 1 Department of Rheumatology, University of Health Sciences, Kartal Dr. Lutfi Kirdar City Hospital, Istanbul 34865, Türkiye; 2 Department of Endocrinology, University of Health Sciences, Sultan 2. Abdulhamid Han Training and Research Hospital, Istanbul 34668, Türkiye; mcan.seno@gmail.com; 3 Department of Internal Medicine, University of Health Sciences, Sancaktepe Sehit Prof. Dr. Ilhan Varank Training and Research Hospital, Istanbul 34785, Türkiye; suleymanbas.2012@gmail.com; 4 Department of Internal Medicine, University of Health Sciences, Eskişehir City Hospital, Eskişehir 26080, Türkiye; cobanhhakan@gmail.com; 5 Department of Neurology, Sultanbeyli State Hospital, Istanbul 34935, Türkiye; ayse_gurbuz90@hotmail.com; 6 Department of Neurology, University of Health Sciences, Sancaktepe Sehit Prof. Dr. Ilhan Varank Training and Research Hospital, Istanbul 34785, Türkiye; drsrkndemir@gmail.com; 7 Department of Internal Medicine, Bahçeşehir University, Göztepe Medical Park Hospital, Istanbul 34865, Türkiye; alpaslantanoglu@yahoo.com

**Keywords:** multiple sclerosis, fingolimod, lymphopenia, infections, hematological changes

## Abstract

*Background and Objectives*: Fingolimod, a sphingosine-1-phosphate receptor modulator, is an effective disease-modifying therapy for relapsing forms of multiple sclerosis (MS). Although lymphopenia is a well-recognized pharmacological effect, its clinical significance remains uncertain. This study evaluated the frequency and severity of fingolimod-associated lymphopenia, longitudinal hematological changes and their relationship with clinical outcomes in a real-world MS cohort. *Materials and Methods*: This retrospective observational study included 143 adults with MS who initiated fingolimod treatment between May 2019 and October 2020. Demographic, clinical and laboratory data were collected at baseline and at 3- and 6-month follow-up visits. Lymphopenia was graded according to the Common Terminology Criteria for Adverse Events version 4.0. Laboratory parameters and infection outcomes were compared according to lymphopenia severity. Logistic regression analysis was performed to identify factors associated with Grade 4 lymphopenia at 6 months. *Results*: At 6 months, lymphopenia was present in 124 patients (86.7%), including Grade 4 lymphopenia in 15 (10.5%). Leukocyte, neutrophil and lymphocyte counts decreased, whereas ALT, AST, GGT, NLR and SII increased significantly during treatment (all *p* < 0.001). Infection frequency did not differ among lymphopenia severity groups (*p* = 0.530). No statistically significant association was detected between Grade 3–4 lymphopenia and infection risk (OR, 1.41; 95% CI, 0.42–4.71; *p* = 0.545). Lower baseline lymphocyte count was independently associated with Grade 4 lymphopenia (adjusted OR 0.078, 95% CI 0.018–0.325; *p* < 0.001). *Conclusions*: Fingolimod-associated lymphopenia was common, with Grade 4 lymphopenia affecting approximately one-tenth of patients. No statistically significant association between lymphopenia severity and infection was detected; however, the small number of Grade 4 cases, low event rate and wide confidence interval limited the statistical power to exclude clinically meaningful differences. These findings should therefore be interpreted cautiously and do not establish the absence of infection risk associated with severe lymphopenia. Regular hematological monitoring and individualized clinical decision-making remain essential, particularly in patients with lower baseline lymphocyte counts.

## 1. Introduction

Multiple sclerosis (MS) is a chronic autoimmune disorder of the central nervous system (CNS), characterized by inflammation, demyelination and neurodegeneration resulting in progressive neurological disability. Over the past two decades, the therapeutic landscape of MS has undergone a paradigm shift with the introduction of highly effective disease-modifying therapies (DMTs), including oral and infusion-based agents, enabling improved disease control and the pursuit of stringent treatment targets such as no evidence of disease activity (NEDA) [1,2,3,4].

Fingolimod, approved in 2010, is the first oral sphingosine-1-phosphate (S1P) receptor modulator introduced for the treatment of relapsing–remitting multiple sclerosis (RRMS). It exerts its therapeutic effect by binding to S1P receptors on lymphocytes, leading to receptor internalization and functional antagonism, thereby preventing lymphocyte egress from lymphoid tissues and limiting the infiltration of autoreactive immune cells into the central nervous system (CNS) [5]. Fingolimod has since been widely used in the management of MS [6].

Despite its well-established efficacy, fingolimod-induced lymphopenia remains a clinically relevant concern, particularly with regard to its potential association with infection risk and long-term safety [2,3]. Although lymphocyte reduction represents an expected pharmacodynamic effect of the drug, substantial interindividual variability exists in both its severity and clinical consequences. Previous studies have reported inconsistent findings regarding the predictors and clinical significance of lymphopenia, and real-world data remain relatively limited. Therefore, a more comprehensive evaluation of lymphopenia patterns and their clinical implications in routine clinical practice is warranted [1,2,3]. Clinical trials with up to three years of follow-up have demonstrated sustained efficacy alongside persistent lymphopenia [7,8]. Although generally manageable, severe lymphopenia (absolute lymphocyte count (ALC) < 200/μL) may require temporary treatment interruption [8,9].

In clinical practice, severe infections related to lymphopenia are relatively uncommon; however, opportunistic infections, including herpes virus reactivation and cryptococcosis, have been reported, raising concerns regarding infection-related adverse outcomes [10]. Identifying patients at increased risk of severe lymphopenia may therefore improve treatment safety and guide monitoring strategies.

Despite the growing number of oral disease-modifying therapies available for MS, optimizing the benefit–risk balance remains a central challenge in clinical practice. While therapeutic advances have enabled the achievement of stringent treatment targets such as NEDA, they have also introduced new challenges related to long-term safety monitoring and individualized risk stratification [11,12]. With the introduction of newer sphingosine-1-phosphate (S1P) receptor modulators, such as siponimod and ponesimod, interest in fingolimod-related hematological changes has increased. However, factors associated with severe lymphopenia, including age, baseline body mass index (BMI), prior disease-modifying therapy exposure and lymphocyte dynamics, remain incompletely understood. Better identification of these factors may help improve patient monitoring and treatment safety in clinical practice [4].

In this current study, we aimed to evaluate the frequency, severity, risk factors, predictors and clinical consequences of fingolimod-associated lymphopenia in a real-world cohort of patients with MS, with a particular focus on identifying risk factors, assessing the likelihood and predictors of accompanying hematological abnormalities, comparing lymphocyte subset profiles between lymphopenic and non-lymphopenic patients and determining the relationship between lymphopenia severity and infection risk.

## 2. Materials and Methods

### 2.1. Study Population

**Figure 1:**
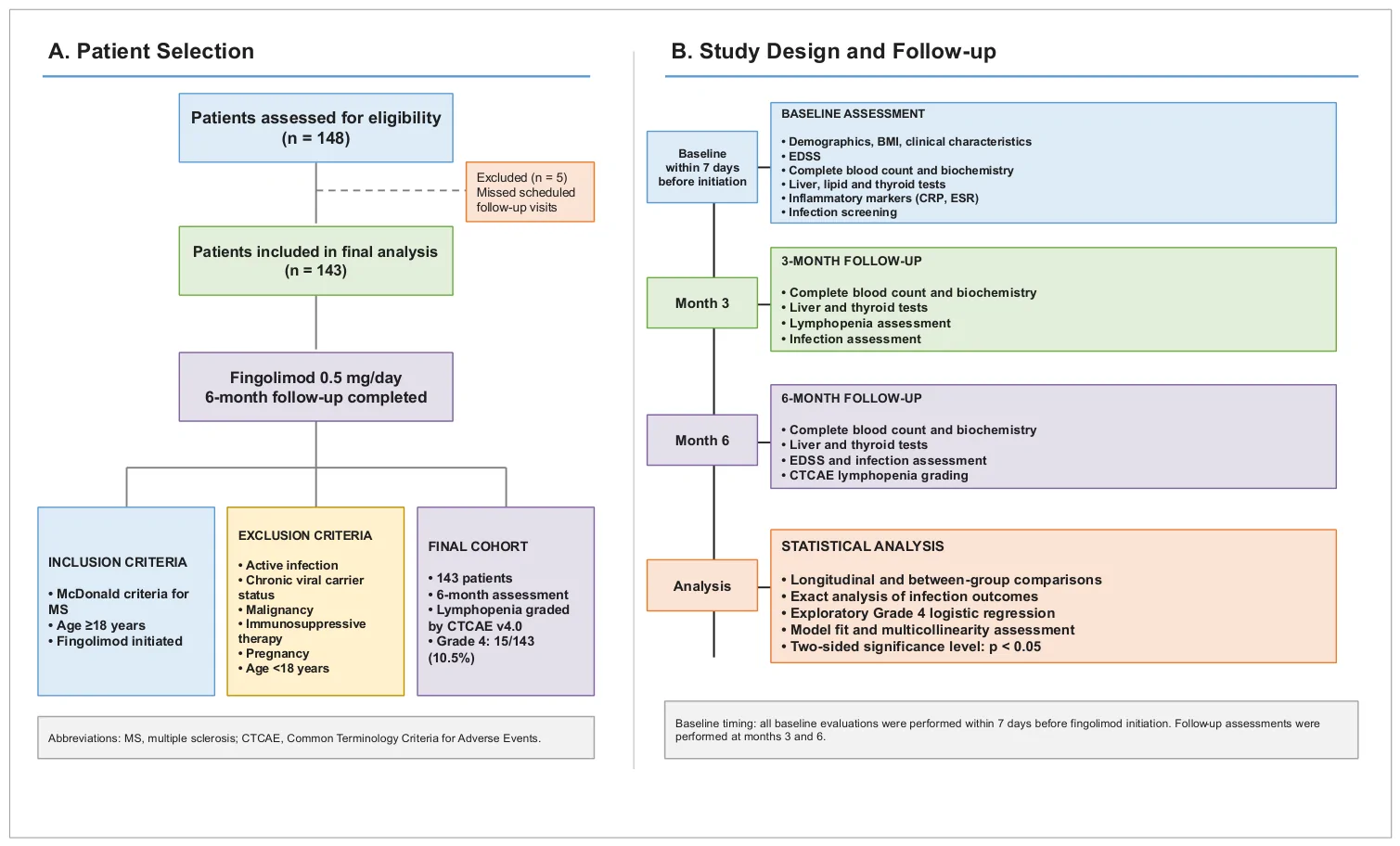
Overview of patient selection, study design, follow-up schedule and outcome assessment. Blue, green, purple, and orange boxes indicate baseline assessment, 3-month follow-up, 6-month follow-up, and exclusion/statistical analysis, respectively. The dashed line indicates exclusion due to incomplete follow-up, whereas solid lines indicate the patient flow and follow-up pathway.

This retrospective observational study included adult patients diagnosed with multiple sclerosis (MS) according to the McDonald criteria who initiated fingolimod treatment between May 2019 and October 2020 at the Neurology Clinic of Sancaktepe Şehit Prof. Dr. İlhan Varank Training and Research Hospital. The study design and patient selection process are illustrated in Figure 1.

Before treatment initiation, all patients underwent routine clinical and laboratory evaluation, including serological screening for varicella-zoster virus (VZV) and herpes simplex virus (HSV) immunoglobulin G (IgG) and immunoglobulin M (IgM), hepatitis B surface antigen (HBsAg), anti-HBs, anti-HCV and anti-HIV. Patients younger than 18 years of age, pregnant women, individuals with active infection, chronic viral carrier status, solid or hematological malignancy or concomitant immunosuppressive therapy were excluded. Fingolimod was initiated at a standard oral dose of 0.5 mg once daily in all eligible patients.

### 2.2. Data Collection

Demographic and clinical variables considered for data collection included age, sex, disease duration, clinical course, previous disease-modifying therapy exposure, smoking status, relapse history, body weight and body mass index (BMI) calculated using the Du Bois formula. However, because of the retrospective nature of the study and incomplete documentation in the available medical records, complete and sufficiently reliable data were not available for disease duration, clinical course, previous disease-modifying therapy exposure, smoking status and relapse history across the study cohort. Therefore, these variables were not included in the comparative baseline analyses or multivariable regression model. BMI data were sufficiently available and were included in the baseline characteristics table and evaluated in the univariable regression analysis. Renal function at treatment initiation was assessed using the Cockcroft–Gault equation to estimate creatinine clearance (CrCl). Laboratory assessments included complete blood count, liver function tests, renal function tests, lipid profile, thyroid function tests and inflammatory markers. Baseline laboratory measurements were obtained within 7 days before fingolimod initiation, followed by reassessments 7 days after treatment initiation and subsequently at 3-month intervals during follow-up. Clinical disability was evaluated using the Expanded Disability Status Scale (EDSS) and infectious complications and laboratory abnormalities occurring during treatment were recorded. Three patients who did not complete the scheduled follow-up assessments were excluded from the final analysis.

### 2.3. Definitions

Lymphopenia severity was classified according to the Common Terminology Criteria for Adverse Events (CTCAE), version 4.0, based on the absolute lymphocyte count (ALC). Grade 1 lymphopenia was defined as an ALC between the lower limit of normal and 800/μL, grade 2 as 500–799/μL, grade 3 as 200–499/μL and grade 4 as <200/μL. Abnormal liver function was defined as aspartate aminotransferase (AST), alanine aminotransferase (ALT) or gamma-glutamyl transferase (GGT) levels exceeding three times the upper limit of normal on two consecutive laboratory assessments.

Clinical outcomes were also recorded, including clinical relapse, as determined by the treating physician based on clinical assessment, radiological disease activity, defined as at least one new or enlarging T2-weighted lesion or a new gadolinium-enhancing lesion compared with baseline magnetic resonance imaging and change in EDSS score assessed between treatment initiation and the 6-month follow-up. Baseline EDSS scores are presented in Table 2 and EDSS scores at the 6-month follow-up are presented in Table 4. During the 6-month follow-up period, no clinical relapses were documented. Brain MRI was performed at the 6-month follow-up after initiation of fingolimod treatment and radiological disease activity was assessed by comparison with baseline MRI findings. Infection was defined as a clinically diagnosed infectious episode documented in the medical record during fingolimod treatment. Infections were categorized according to their clinical characteristics and when available, microbiological findings. Opportunistic infections were defined as infections caused by pathogens or occurring in clinical settings typically associated with impaired cellular immune function, including herpesvirus reactivation and other opportunistic pathogens documented during follow-up.

Treatment discontinuation was defined as permanent cessation of fingolimod therapy for any reason during the study follow-up period. Temporary treatment interruption was recorded separately when applicable.

### 2.4. Ethics Statement

The study protocol was approved by the Sancaktepe Şehit Prof. Dr. İlhan Varank Training and Research Hospital Clinical Research Ethics Committee (Approval No: E-46059653-050.04-312755333, Approval Date: 27 April 2026). The study was conducted in accordance with the ethical principles of the Declaration of Helsinki. Owing to the retrospective design of the study and the use of anonymized clinical data, the requirement for written informed consent was waived by the Ethics Committee.

### 2.5. Statistical Analysis

Statistical analyses were performed using IBM SPSS Statistics for Windows, Version 25.0 (IBM Corp., Armonk, NY, USA). Continuous variables were presented as mean ± standard deviation (SD) when normally distributed and as median (interquartile range [IQR]) when non-normally distributed. Categorical variables were expressed as frequencies and percentages. The distribution of continuous variables was assessed using visual methods and the Kolmogorov–Smirnov and Shapiro–Wilk tests.

Comparisons between two independent groups were performed using the Independent T Test for normally distributed continuous variables and the Mann–Whitney U Test for non-normally distributed continuous variables. Comparisons among three independent groups were performed using one-way analysis of variance (ANOVA) for normally distributed variables and the Kruskal–Wallis test for non-normally distributed variables. When a statistically significant omnibus difference was detected, Bonferroni-adjusted post hoc comparisons were performed following ANOVA, whereas pairwise Mann–Whitney U tests were performed following the Kruskal–Wallis test. Repeated measurements were compared using repeated-measures ANOVA for normally distributed variables and the Friedman test for non-normally distributed variables.

Categorical variables were compared using the chi-square test. When expected cell counts were insufficient for the chi-square approximation, exact tests were used. Because of sparse expected cell counts, the frequency of any infection across the three lymphopenia groups was compared using the Fisher–Freeman–Halton exact test with Monte Carlo estimation based on 10,000 sampled tables. To provide an exploratory effect estimate for infection, patients with Grade 3–4 lymphopenia were compared with patients without lymphopenia or with Grade 1–2 lymphopenia in a binary analysis. Grade 3–4 lymphopenia was considered a severe lymphopenia category for these exploratory analyses because of the limited number of Grade 4 events. The association was reported as an odds ratio (OR) with a 95% confidence interval (CI) and statistical inference was based on the two-sided Fisher’s exact test. Infection subtypes were presented descriptively because of the small number of events.

Univariable binary logistic regression analyses were performed to explore factors associated specifically with Grade 4 lymphopenia at the 6-month follow-up. Grade 4 lymphopenia was coded as 1, whereas no lymphopenia or Grade 1–3 lymphopenia was coded as 0. Age, sex, BMI, baseline lymphocyte count and baseline serum creatinine were evaluated in the univariable analyses. Because only 15 patients developed Grade 4 lymphopenia, the multivariable model was restricted to age, sex and baseline lymphocyte count to reduce the risk of overfitting. BMI and baseline serum creatinine were therefore retained only in the univariable analyses. Disease duration, clinical course, previous disease-modifying therapy exposure, smoking status and relapse history were not reliably available in the dataset and were not included in the regression analyses.

Model fit was evaluated using the omnibus likelihood-ratio chi-square test and the Hosmer–Lemeshow goodness-of-fit test. Nagelkerke R^2^ was reported as a measure of explained variation. Classification performance was summarized using overall classification accuracy, sensitivity and specificity at a probability cutoff of 0.50. Multicollinearity among the variables included in the multivariable model was assessed using tolerance values and variance inflation factors (VIFs). Tolerance values <0.20 or VIF values > 5 were considered indicative of potentially relevant multicollinearity. Because SPSS does not provide VIF statistics directly within binary logistic regression, collinearity diagnostics were obtained from an auxiliary linear regression model containing the same predictors and were used solely to assess multicollinearity. Regression results were reported as ORs with 95% CIs. All tests were two-sided, and *p* < 0.05 was considered statistically significant.

## 3. Results

### 3.1. Patient Characteristics and Longitudinal Changes

A total of 143 patients were included in the final analysis. The mean age was 35.50 ± 9.62 years, 90 patients (62.9%) were female and the mean BMI was 26.52 ± 2.29 kg/m^2^. Baseline demographic, clinical, biochemical and hematological characteristics and their changes during follow-up are presented in Table 1.

Serum glucose, creatinine, lipid parameters, CRP, ESR, TSH, platelet count, MPV and PCT did not change significantly during the 6-month follow-up (all *p* > 0.05). In contrast, ALT, AST and GGT levels increased significantly from baseline to the 3- and 6-month follow-ups (all *p* < 0.001; Table 1).

Marked changes in peripheral blood cell counts were observed during fingolimod treatment. Leukocyte, neutrophil and lymphocyte counts decreased significantly during follow-up (all *p* < 0.001). The greatest reduction was observed in lymphocyte count, which decreased from a median of 1.52 (1.11–2.00) × 10^3^/µL at baseline to 0.65 (0.44–0.80) × 10^3^/µL at 3 months and 0.56 (0.43–0.78) × 10^3^/µL at 6 months. Monocyte count, hemoglobin, and hematocrit did not change significantly (all *p* > 0.05). NLR and SII increased significantly during follow-up (both *p* < 0.001; Table 1).

### 3.2. Findings According to Lymphopenia Status

At baseline, lymphopenia was present in 19 patients (13.3%). Patients with baseline lymphopenia were older than those without lymphopenia (39.95 ± 11.90 vs. 34.81 ± 9.04 years; *p* = 0.010). Baseline BMI did not differ between the groups (26.91 ± 2.44 vs. 26.46 ± 2.27 kg/m^2^; *p* = 0.429). Patients with baseline lymphopenia also had lower leukocyte (*p* < 0.001), lymphocyte (*p* < 0.001), monocyte (*p* = 0.006), platelet (*p* = 0.025) and PCT values (*p* = 0.035). No statistically significant differences were observed in sex, EDSS score or the remaining biochemical and hematological parameters (Table 2).

**Table 2:** Baseline characteristics compared according to lymphopenia status.

	Lymphopenia Present (n = 19, 13.3%)	Lymphopenia Absent (n = 124, 86.7%)	*p*
**Sex (Male/Female)**	7 (36.8%)/12 (63.2%)	46 (37.1%)/78 (62.9%)	0.983 ^c^
**Age (years)**	39.95 ± 11.90 ^a^	34.81 ± 9.04 ^a^	0.010 ^d,^*
**BMI (kg/m^2^)**	26.91 ± 2.44 ^a^	26.46 ± 2.27 ^a^	0.429 ^d^
**EDSS**	3 (3–4) ^b^	3 (2.50–3.50) ^b^	0.186 ^e^
**Glucose (mg/dL)**	92 (86–100) ^b^	89 (83–97.25) ^b^	0.211 ^e^
**Creatinine (mg/dL)**	0.73 ± 0.11 ^a^	0.75±0.15 ^a^	0.594 ^d^
**ALT (U/L)**	17.80 (12–25) ^b^	16 (11–23) ^b^	0.761 ^e^
**AST (U/L)**	17 (15–19.90) ^b^	16.50 (14–20) ^b^	0.780 ^e^
**GGT (U/L)**	12 (9–22) ^b^	15 (10–22) ^b^	0.537 ^e^
**LDL cholesterol (mg/dL)**	106.40 (73.75–125.25) ^b^	114.50 (90–137.50) ^b^	0.358 ^e^
**HDL cholesterol (mg/dL)**	55.50 (43.75–61.21) ^b^	54 (45–65.75) ^b^	0.690 ^e^
**Total cholesterol (mg/dL)**	180 (147.61–203.25) ^b^	185 (161–223) ^b^	0.503 ^e^
**Triglyceride (mg/dL)**	93 (57.68–155.75) ^b^	100 (74–147.50) ^b^	0.653 ^e^
**Leukocyte (×10^3^/µL)**	4.42 (3.50–5.37) ^b^	6.65 (5.49–8.33) ^b^	<0.001 ^e,^*
**Neutrophil (×10^3^/µL)**	3.25 (2.34–4.65) ^b^	3.99 (3.24–5.36) ^b^	0.161 ^e^
**Lymphocyte (×10^3^/µL)**	0.76 (0.50–0.92) ^b^	1.61 (1.24–2.04) ^b^	<0.001 ^e,^*
**Monocyte (×10^3^/µL)**	0.34 (0.28–0.46) ^b^	0.50 (0.35–0.63) ^b^	0.006 ^e,^*
**Hemoglobin (g/dL)**	13.08 ± 1.73 ^a^	13.59 ± 1.67 ^a^	0.283 ^d^
**Hematocrit (%)**	39.85 ± 4.81 ^a^	40.80 ± 4.44 ^a^	0.444 ^d^
**Platelets (×10^3^/µL)**	218.08 ± 47.41 ^a^	256.60 ± 62.83 ^a^	0.025 ^d,^*
**MPV (fL)**	9.43 ± 1.50 ^a^	9.57 ± 1.33 ^a^	0.708 ^d^
**PCT (%)**	0.20 ± 0.04 ^a^	0.24 ± 0.06 ^a^	0.035 ^d,^*
**CRP (mg/dL)**	1.55 (0.65–3.14) ^b^	1.14 (0.20–2) ^b^	0.339 ^e^
**ESR (mm/h)**	4 (2.75–18.25) ^b^	9 (5–17.25) ^b^	0.294 ^e^
**TSH (mIU/L)**	1.51 (1.13–1.90) ^b^	1.43 (0.99–2.15) ^b^	0.990 ^e^

^a^: Mean ± Standard deviation (SD); ^b^: Median (IQR: Interquartile Range); ^c^: Chi-Square Test; ^d^: Independent T Test; ^e^: Mann–Whitney U Test. *: Statistically significant (*p* < 0.05).

At the 3-month follow-up, lymphopenia was present in 123 patients (86.0%). Male sex was more frequent among patients without lymphopenia than among those with lymphopenia (60.0% vs. 33.3%; *p* = 0.022). Patients with lymphopenia had lower creatinine (*p* = 0.019), triglyceride (*p* = 0.007), leukocyte (*p* < 0.001), lymphocyte (*p* < 0.001) and TSH levels (*p* = 0.022). Age, BMI, EDSS score, liver enzymes, inflammatory markers and the remaining laboratory parameters did not differ significantly between the groups (Table 3).

**Table 3:** Three-month follow-up characteristics compared according to lymphopenia status.

	Lymphopenia Present (n = 123, 86%)	Lymphopenia Absent (n = 20, 14%)	*p*
**Sex (Male/Female)**	41 (33.3%)/82 (66.7%)	12 (60%)/8 (40%)	0.022 ^c,^*
**Age (years)**	35.84 ± 9.88 ^a^	33.45 ± 7.7 ^a^	0.305 ^d^
**BMI (kg/m^2^)**	26.55 ± 2.34 ^a^	26.28 ± 1.96 ^a^	0.622 ^d^
**Glucose (mg/dL)**	90.92 ± 11.12 ^a^	92.61 ± 13.33 ^a^	0.615 ^d^
**Creatinine (mg/dL)**	0.72 (0.63–0.81) ^b^	0.83 (0.68–1,04) ^b^	0.019 ^e,^*
**ALT (U/L)**	23 (13.25–38) ^b^	22 (16.50–39.50) ^b^	0.665 ^e^
**AST (U/L)**	20 (16–26) ^b^	20 (16.50–27) ^b^	0.845 ^e^
**GGT (U/L)**	18 (12–34) ^b^	24 (20–40) ^b^	0.174 ^e^
**LDL cholesterol (mg/dL)**	114 (93–138.50) ^b^	107 (92.05–190.35) ^b^	0.569 ^e^
**HDL cholesterol (mg/dL)**	57.76 ± 15.46 ^a^	52.90 ± 13.02 ^a^	0.401 ^d^
**Total cholesterol (mg/dL)**	195 (175.25–220.50) ^b^	193 (165–273) ^b^	0.675 ^e^
**Triglyceride (mg/dL)**	91 (63–118) ^b^	161 (129–196) ^b^	0.007 ^e,^*
**Leukocyte (×10^3^/µL)**	4.75 ± 1.75 ^a^	6.03 ± 1.77 ^a^	<0.001 ^d,^*
**Neutrophil (×10^3^/µL)**	3.46 ± 1.14 ^a^	3.83 ± 0.84 ^a^	0.306 ^d^
**Lymphocyte (×10^3^/µL)**	0.58 (0.42–0.77) ^b^	1.44 (1.21–1.81) ^b^	<0.001 ^e,^*
**Monocyte (×10^3^/µL)**	0.44 (0.33–0.52) ^b^	0.56 (0.45–0.70) ^b^	0.051 ^e^
**Hemoglobin (g/dL)**	13.53 ± 1.58 ^a^	14.35 ± 1.44 ^a^	0.105 ^d^
**Hematocrit (%)**	40.39 ± 4.26 ^a^	42.09 ± 4.06 ^a^	0.212 ^d^
**Platelets (×10^3^/µL)**	253.75 ± 56.71 ^a^	254.11 ± 42.89 ^a^	0.984 ^d^
**MPV (fL)**	9.61 ± 1.29 ^a^	9.30 ±1.32 ^a^	0.468 ^d^
**PCT (×%)**	0.24 (0.20–0.28) ^b^	0.23 (0.22–0.25) ^b^	0.611 ^e^
**CRP (mg/dL)**	0.64 (0.21–2) ^b^	2 (0.40–3.52) ^b^	0.139 ^e^
**ESR (mm/h)**	9 (4–19) ^b^	4 (2.50–13) ^b^	0.262 ^e^
**TSH (mIU/L)**	1.89 ± 0.82 ^a^	1.15 ± 0.40 ^a^	0.022 ^d,^*

^a^: Mean ± Standard deviation (SD); ^b^: Median (IQR: Interquartile Range); ^c^: Chi-Square Test; ^d^: Independent T Test; ^e^: Mann-Whitney U Test. *: Statistically significant (*p* < 0.05).

At the 6-month follow-up, lymphopenia was present in 124 patients (86.7%). Male sex was more frequent among patients without lymphopenia than among those with lymphopenia (63.2% vs. 33.1%; *p* = 0.011). Patients with lymphopenia had lower creatinine (*p* = 0.025), GGT (*p* = 0.010), triglyceride (*p* = 0.022), leukocyte (*p* < 0.001), neutrophil (*p* = 0.002) and lymphocyte levels (*p* < 0.001), whereas HDL cholesterol was higher (*p* = 0.036). Age, BMI, EDSS score, inflammatory markers, thyroid function and the remaining biochemical and hematological parameters did not differ significantly between the groups (Table 4). Given the number of comparisons performed, the isolated differences in creatinine, GGT, HDL cholesterol and triglycerides should be regarded as exploratory.

**Table 4:** Six-month follow-up characteristics compared according to lymphopenia status.

	Lymphopenia Present (n = 124, 86.7%)	Lymphopenia Absent (n = 19, 13.3%)	*p*
**Sex (Male/Female)**	41 (33.1%)/83 (66.9%)	12 (63.2%)/7 (36.8%)	0.011 ^c,^*
**Age (years)**	35.23 ± 9.91 ^a^	37.32 ± 7.41 ^a^	0.380 ^d^
**BMI (kg/m^2^)**	26.46 ± 2.28 ^a^	26.86 ± 2.38 ^a^	0.481 ^d^
**EDSS**	2.50 (2–3) ^b^	2.50 (2.50–3) ^b^	0.372 ^e^
**Glucose (mg/dL)**	89 (84–96) ^b^	94 (84–104) ^b^	0.099 ^e^
**Creatinine (mg/dL)**	0.71 (0.64–0.82) ^b^	0.79 (0.73–0.98) ^b^	0.025 ^e,^*
**ALT (U/L)**	26.50 (16.75–45) ^b^	27 (11–53) ^b^	0.712 ^e^
**AST (U/L)**	21 (16–28) ^b^	27 (13–32) ^b^	0.890 ^e^
**GGT (U/L)**	21 (13–35) ^b^	37 (26–58) ^b^	0.010 ^e,^*
**LDL cholesterol (mg/dL)**	112 (88–143) ^b^	128 (112.75–139.82) ^b^	0.163 ^e^
**HDL cholesterol (mg/dL)**	57 (50–71) ^b^	49.40 (39.50–60.75) ^b^	0.036 ^e,^*
**Total cholesterol (mg/dL)**	197.17 ± 53.69 ^a^	199.75 ± 61.45 ^a^	0.870 ^d^
**Triglyceride (mg/dL)**	86 (66.95–136) ^b^	175.50 (81.15–241.25) ^b^	0.022 ^e,^*
**Leukocyte (×10^3^/µL)**	4.60 ± 1.50 ^a^	6.83 ± 2.19 ^a^	<0.001 ^d,^*
**Neutrophil (×10^3^/µL)**	3.25 (2.6–4.16) ^b^	4.54 (3.56–5.63) ^b^	0.002 ^e,^*
**Lymphocyte (×10^3^/µL)**	0.53 (0.40–0.66) ^b^	1.31 (1.16–1.78) ^b^	<0.001 ^e,^*
**Monocyte (×10^3^/µL)**	0.44 ± 0.13 ^a^	0.48 ± 0.14 ^a^	0.223 ^d^
**Hemoglobin (g/dL)**	13.44 ± 1.63 ^a^	14.07 ± 1.82 ^a^	0.133 ^d^
**Hematocrit (%)**	40.13 ± 4.29 ^a^	41.61 ± 4.89 ^a^	0.177 ^d^
**Platelets (×10^3^/µL)**	252.56 ± 57.52 ^a^	280.10 ± 61.43 ^a^	0.060 ^d^
**MPV (fL)**	9.62±1.32 ^a^	9.37 ± 1.22 ^a^	0.452 ^d^
**PCT (%)**	0.23 (0.20–0.27) ^b^	0.25 (0.22–0.28) ^b^	0.152 ^e^
**CRP (mg/dL)**	0.83 (0.51–2) ^b^	0.89 (0.20–2.85) ^b^	0.946 ^e^
**ESR (mm/h)**	8 (5–18) ^b^	10.50 (8.50–24.50) ^b^	0.128 ^e^
**TSH (mIU/L)**	1.7 ± 0.81 ^a^	1.72 ± 0.50 ^a^	0.933 ^d^

^a^: Mean ± Standard deviation (SD); ^b^: Median (IQR: Interquartile Range); ^c^: Chi-Square Test; ^d^: Independent T Test; ^e^: Mann-Whitney U Test. *: Statistically significant (*p* < 0.05).

### 3.3. Findings According to Lymphopenia Severity and Infection Outcomes

At the 6-month follow-up, 19 patients (13.3%) had no lymphopenia, 75 (52.4%) had Grade 1–2 lymphopenia and 49 (34.3%) had Grade 3–4 lymphopenia. Clinical and laboratory findings according to lymphopenia severity are summarized in Table 5.

Creatinine (*p* = 0.013), GGT (*p* = 0.032) and HDL cholesterol (*p* = 0.027) differed significantly across the three groups. Post hoc analyses showed that patients in the Grade 3–4 lymphopenia group had lower creatinine and higher HDL cholesterol levels compared with those without lymphopenia, whereas GGT levels were higher in patients without lymphopenia than in both lymphopenia groups. Given the exploratory nature of these univariate comparisons and the large number of statistical tests performed, these biochemical findings should be interpreted cautiously and considered hypothesis-generating rather than confirmatory. Leukocyte (*p* < 0.001), neutrophil (*p* = 0.003) and lymphocyte counts (*p* < 0.001) progressively decreased with increasing lymphopenia severity. Hemoglobin (*p* = 0.015) and hematocrit (*p* = 0.007) also differed significantly across the groups and were lowest in patients with Grade 3–4 lymphopenia. Age, BMI, EDSS score, platelet count, inflammatory markers, thyroid function and the remaining laboratory parameters did not differ significantly among the three groups.

During the 6-month follow-up, infections were documented in 12 of 143 patients (8.4%). No infections occurred among patients without lymphopenia, whereas infections were recorded in 7 of 75 patients (9.3%) with Grade 1–2 lymphopenia and 5 of 49 patients (10.2%) with Grade 3–4 lymphopenia. The frequency of any infection did not differ significantly across the three groups (Fisher–Freeman–Halton exact test, Monte Carlo *p* = 0.530). In an exploratory binary comparison, the OR for any infection among patients with Grade 3–4 lymphopenia versus those without lymphopenia or with Grade 1–2 lymphopenia was 1.41 (95% CI, 0.42–4.71; Fisher’s exact *p* = 0.545). Given the small number of infection events, the wide confidence interval indicates substantial uncertainty around the effect estimate and does not exclude either a clinically meaningful increase or decrease in infection risk. Infection subtypes are presented descriptively in Table 5.

### 3.4. Factors Associated with Grade 4 Lymphopenia

Grade 4 lymphopenia occurred in 15 of 143 patients (10.5%). An exploratory logistic regression analysis was performed to identify factors associated specifically with Grade 4 lymphopenia at the 6-month follow-up (Table 6).

In the univariable analyses, lower baseline lymphocyte count was significantly associated with Grade 4 lymphopenia (OR per 1 × 10^3^/µL increase, 0.084; 95% CI 0.021–0.335; *p* < 0.001). Age (OR 0.991, 95% CI 0.936–1.049; *p* = 0.742), female sex (OR 2.564, 95% CI 0.689–9.541; *p* = 0.160), BMI (OR 0.907, 95% CI 0.704–1.170; *p* = 0.453) and baseline creatinine (OR 0.008, 95% CI <0.001–1.048; *p* = 0.052) were not statistically significant.

In the multivariable model including age, sex and baseline lymphocyte count, lower baseline lymphocyte count remained independently associated with Grade 4 lymphopenia (adjusted OR per 1 × 10^3^/µL increase, 0.078; 95% CI 0.018–0.325; *p* < 0.001). Age (adjusted OR 0.989, 95% CI 0.934–1.047; *p* = 0.693) and female sex (adjusted OR 2.883, 95% CI 0.711–11.687; *p* = 0.138) were not independently associated with Grade 4 lymphopenia.

The multivariable model was statistically significant compared with the intercept-only model (χ^2^ = 20.493, df = 3; *p* < 0.001) and had a Nagelkerke R^2^ of 0.273. The Hosmer–Lemeshow test indicated no evidence of poor model fit (χ^2^ = 5.520, df = 8; *p* = 0.701). No relevant multicollinearity was detected among the included predictors (tolerance range, 0.978–0.998; VIF range, 1.002–1.023). At a probability cutoff of 0.50, the model achieved an overall classification accuracy of 90.2%, with a sensitivity of 20.0% and a specificity of 98.4%. However, the high overall accuracy was largely attributable to the predominance of patients without Grade 4 lymphopenia. Given the limited number of Grade 4 events, the model’s classification performance and regression estimates should be interpreted as exploratory and with caution.

Exploratory infection analysis: Patients with Grade 3–4 lymphopenia were compared with patients without severe lymphopenia (no lymphopenia/Grade 1–2). The OR for any infection was 1.41 (95% CI 0.42–4.71; Fisher’s exact *p* = 0.545). Subtype-specific effect estimates were not calculated because of the small number of infection events. CI: Confidence interval; OR: Odds Ratio.

## 4. Discussion

In the present study, fingolimod-associated lymphopenia was highly prevalent with 124 patients (86.7%) developing grade 1–4 lymphopenia after treatment initiation. Grade 4 lymphopenia occurred in approximately 10.5% of patients. No statistically significant association was detected between lymphopenia severity and infection frequency, disease activity or serious adverse events. However, these findings should be interpreted cautiously given the limited number of Grade 4 cases and clinical events. Because of the retrospective design, treatment interruption or dose modification based on lymphocyte counts was not systematically implemented.

Post-marketing safety data indicate that reduced lymphocyte counts remain a commonly reported adverse event with fingolimod. Pharmacovigilance analyses of European adverse event reporting systems have identified lymphopenia among the most frequently reported adverse reactions in fingolimod-treated patients with MS [12,13]. Consistent with these observations, our findings confirm that lymphopenia is a frequent hematological effect of fingolimod in routine clinical practice.

The clinical relevance of fingolimod-associated lymphopenia, particularly its relationship with infection risk, remains incompletely defined. Previous phase III clinical trials did not demonstrate a strong correlation between lymphocyte counts and overall infection incidence while opportunistic infections such as progressive multifocal leukoencephalopathy remained rare [14]. In our cohort, no statistically significant association was observed between lymphopenia severity and infection frequency. Nevertheless, the relatively small number of infectious events limits the precision of this estimate; therefore, the absence of statistical significance should not be interpreted as evidence that no clinically relevant association exists. Continuous monitoring of absolute lymphocyte counts (ALC) may nevertheless facilitate appropriate clinical surveillance and individualized management.

Real-world studies have reported Grade 4 lymphopenia in approximately 15–20% of fingolimod-treated patients in Western populations, whereas an early retrospective Japanese cohort reported rates of up to 29% [15]. In comparison, the incidence in our cohort was approximately 10%. Differences in patient characteristics, laboratory monitoring schedules, assessment methods and treatment management may partly explain these variations. Data from long-term progressive MS cohorts also suggest that the proportion of patients developing Grade 4 lymphopenia does not necessarily increase with prolonged fingolimod exposure, although continued ALC monitoring remains important for identifying persistent or recurrent severe lymphopenia.

Previous studies have reported several potential predictors of severe fingolimod-associated lymphopenia, including lower baseline lymphocyte count, lower BMI and previous interferon-beta exposure [16]. In our cohort, lower baseline lymphocyte count was independently associated with Grade 4 lymphopenia in the exploratory multivariable analysis, whereas age and sex were not significantly associated with the outcome. BMI and baseline serum creatinine were also not significantly associated with Grade 4 lymphopenia in univariable analyses. Given that only 15 Grade 4 events were observed, these analyses had limited statistical power and were susceptible to imprecision. Accordingly, the association between lower baseline lymphocyte count and Grade 4 lymphopenia should be considered exploratory and requires confirmation in larger prospective cohorts.

No statistically significant association was detected between lymphopenia severity and the assessed measures of disease activity. Peripheral lymphocyte counts have not consistently been shown to predict clinical outcomes, whereas emerging evidence suggests that specific lymphocyte subsets, including CD4-positive T cells and thymic emigrants, may provide more informative measures of immune status during fingolimod therapy. Several laboratory parameters, including creatinine, GGT, HDL cholesterol and triglycerides, differed significantly across lymphopenia severity groups in univariate analyses. However, because numerous comparisons were performed without formal adjustment for multiple testing, these findings should be regarded as exploratory and hypothesis-generating rather than confirmatory.

The overall infection incidence in our cohort was 8.4% which was lower than that reported in some clinical trials (approximately 51–53%) but comparable to rates reported in real-world observational cohorts [17]. Long-term safety studies have not consistently demonstrated an excess of serious infections with fingolimod compared with placebo. In our cohort, only one patient (approximately 0.7%) developed herpes zoster with no clear association with Grade 4 lymphopenia. Previous studies have similarly suggested that herpes zoster reactivation may not correlate directly with ALC [13]. Pre-treatment herpes zoster vaccination, administered to 17.4% of our patients, may also have contributed to the low incidence observed. Findings from the INFORMS study and post-marketing surveillance have likewise indicated that serious infections are uncommon during fingolimod treatment [7,8].

Among patients who developed Grade 4 lymphopenia, treatment was continued with individualized dosing adjustments. Six patients developed Grade 4 lymphopenia at month 3 and received alternate-day administration, whereas the remaining nine patients were identified at month 6. These observations illustrate the individualized treatment strategies used in clinical practice; however, they should not be interpreted as evidence that treatment discontinuation can safely be avoided solely on the basis of lymphocyte counts [18]. Decisions regarding dose modification or treatment interruption should consider the severity and persistence of lymphopenia together with the patient’s overall clinical status and the potential risk of disease rebound following fingolimod discontinuation.

Hepatic safety was generally acceptable, with ALT or AST levels ≥ 3× the upper limit of normal observed in 8.7% of patients, comparable to rates reported in clinical trials (8–8.5%). The treatment discontinuation rate was 14.5%, within the range reported in previous studies (10.6–43.8%), and Grade 4 lymphopenia was not identified as a major determinant of treatment cessation in our cohort.

This study has several limitations. Its retrospective, single-center design and the non-standardized timing of laboratory assessments may have influenced the observed dynamics of lymphopenia. The relatively small number of Grade 4 cases and clinical events limited the statistical power to detect clinically meaningful associations with infection and other outcomes. The six-month follow-up was adequate for evaluating early lymphopenia dynamics but insufficient to assess late or rare infectious complications, particularly opportunistic infections. In addition, the exploratory multivariable analysis was restricted to age, sex and baseline lymphocyte count because of the limited number of Grade 4 events, whereas BMI and baseline serum creatinine were evaluated only in univariable analyses. Finally, the large number of univariate comparisons without formal correction for multiple testing increases the possibility of type I error. Larger, prospective, multicenter studies with standardized follow-up and more comprehensive immune and clinical assessments are needed to clarify the long-term clinical implications and predictors of severe fingolimod-associated lymphopenia.

Overall, fingolimod treatment was associated with lymphopenia in the majority of patients, with Grade 4 lymphopenia occurring in approximately 10.5% of this Turkish MS cohort. No statistically significant association was identified between lymphopenia severity and infection frequency or disease activity; however, the limited number of severe lymphopenia cases and clinical events precludes definitive conclusions regarding these relationships. These findings underscore the importance of continued ALC monitoring and individualized clinical management and highlight the need for larger prospective studies to better define the clinical significance and long-term consequences of severe fingolimod-associated lymphopenia.

## 5. Conclusions

This real-world study found that fingolimod-associated lymphopenia was highly prevalent among Turkish patients with MS, with Grade 4 lymphopenia occurring in approximately 10.5% of patients. We did not detect a statistically significant association between lymphopenia severity and infection risk or other adverse clinical outcomes. However, given the small number of grade 4 lymphopenia cases (n = 15) and the low event rates throughout follow-up, the study was underpowered to exclude clinically relevant risks and these null findings should be interpreted as a failure to detect an association rather than as evidence of no risk. Consistent with the current fingolimod prescribing information, the occurrence of severe (Grade 4) lymphopenia necessitates careful clinical evaluation and when indicated, temporary treatment interruption to mitigate potential infectious complications, irrespective of the absence of a statistically significant association in this cohort. Therefore, regular hematological monitoring and individualized clinical decision-making, guided by the approved product label, remain essential for the safe management of patients receiving fingolimod.

## Figures and Tables

**Table 1:** Laboratory and clinical parameters at baseline, 3-month and 6-month follow-up.

	All Patients (n = 143)			
**Age (years)**	35.50 ± 9.62 ^a^			
**Sex (Male/Female)**	53 (37.1%)/90 (62.9%)			
**BMI (kg/m^2^)**	26.52 ± 2.29 ^a^			
	**Baseline**	**3-month follow-up**	**6-month follow-up**	***p***
**Glucose (mg/dL)**	89 (83–98) ^b^	91 (83–97) ^b^	89 (84–96) ^b^	0.820 ^c^
**Creatinine (mg/dL)**	0.74 (0.65–0.83) ^b^	0.72 (0.63–0.82) ^b^	0.73 (0.65–0.84) ^b^	0.149 ^c^
**ALT (U/L)**	16 (11–23) ^b^	23 (14–38.50) ^b^	27 (16–45.50) ^b^	<0.001 ^c,^*
**AST (U/L)**	17 (14–20) ^b^	20 (16.25–25.75)	21 (16–29.25) ^b^	<0.001 ^c,^*
**GGT (U/L)**	15 (10–22) ^b^	21 (13–34) ^b^	23 (15–41) ^b^	<0.001 ^c,^*
**LDL cholesterol** **(mg/dL)**	113.10 (90–135.75) ^b^	112.56 (93.5–140.25) ^b^	115 (89–141.50) ^b^	0.396 ^c^
**HDL cholesterol (mg/dL)**	54 (44.75–65) ^b^	55 (44.50–66) ^b^	56 (49.40–71) ^b^	0.316 ^c^
**Total cholesterol (mg/dL)**	185 (160–222.50) ^b^	195 (175–223) ^b^	195.50 (161.50–223) ^b^	0.142 ^c^
**Triglyceride (mg/dL)**	100 (70.50–144) ^b^	94 (69–126) ^b^	87 (68–147) ^b^	0.811 ^c^
**Leukocyte (×10^3^/µL)**	6.54 (5.07–8.02) ^b^	4.78 (3.88–5.76) ^b^	4.71 (3.50–5.91) ^b^	<0.001 ^c,^*
**Neutrophil (×10^3^/µL)**	3.96 (3.18–5.34) ^b^	3.32 (2.70–4.12) ^b^	3.39 (2.62–4.51) ^b^	<0.001 ^c,^*
**Lymphocyte (×10^3^/µL)**	1.52 (1.11–2) ^b^	0.65 (0.44–0.80) ^b^	0.56 (0.43–0.78) ^b^	<0.001 ^c,^*
**Monocyte (×10^3^/µL)**	0.49 (0.33–0.61) ^b^	0.46 (0.35–0.56) ^b^	0.44 (0.36–0.54) ^b^	0.616 ^c^
**Hemoglobin (g/dL)**	13.52 ± 1.68 ^a^	13.62 ± 1.58 ^a^	13.54 ± 1.67 ^a^	0.139 ^d^
**Hematocrit (%)**	40.68 ± 4.48 ^a^	40.57 ± 4.25 ^a^	40.35 ± 4.40 ^a^	0.139 ^d^
**Platelets (×10^3^/µL)**	242 (204.50–286.10) ^b^	249 (211–286) ^b^	246 (221–283.50) ^b^	0.226 ^c^
**MPV (fL)**	9.55 ± 1.35 ^a^	9.57 ± 1.29 ^a^	9.58 ± 1.30 ^a^	0.891 ^d^
**PCT (%)**	0.23 (0.20–0.28) ^b^	0.23 (0.20–0.27) ^b^	0.24 (0.20–0.27) ^b^	0.722 ^c^
**CRP (mg/dL)**	1.14 (0.32–2) ^b^	0.72 (0.25–2.01) ^b^	0.83 (0.28–2) ^b^	0.390 ^c^
**ESR (mm/h)**	9 (4.50–17.75) ^b^	8.50 (3.75–18.25) ^b^	8 (5–18) ^b^	0.096 ^c^
**TSH (mIU/L)**	1.48 (1.01–2.01) ^b^	1.71 (1.27–2.37) ^b^	1.61 (1.12–2.19) ^b^	0.141 ^c^
**NLR**	2.52 (1.84–3.46) ^b^	5.65 (3.92–7.86) ^b^	5.46 (4–8.29) ^b^	<0.001 ^c,^*
**Systemic Immune-Inflammation Index (NLRxplatelet count)**	617.95 (457.05–981.11) ^b^	1383.07 (919.57–2066.94) ^b^	1438.15 (979.70–2092.11) ^b^	<0.001 ^c,^*

^a^: Mean ± Standard deviation (SD); ^b^: Median (IQR: Interquartile Range); ^c^: Friedman Test; ^d^: Repeated-measures ANOVA Test. *: Statistically significant (*p* < 0.05).

**Table 5:** Six-month follow-up characteristics compared according to the severity of lymphopenia.

	Lymphopenia Absent (n = 19, 13.3%)	Lymphopenia, Grades 1–2 (n = 75, 52.4%)	Lymphopenia, Grades 3–4 (n = 49, 34.3%)	*p*
**Sex (Male/Female)**	12 (63.2%) ^k^ 7 (36.8%) ^l^	29 (38.7%)/ 46 (61.3%)	12 (24.5%) ^k^ 37 (75.5%) ^l^	0.011 ^c,^*
**Age (years)**	37.32 ± 7.41 ^a^	34.89 ± 10.05 ^a^	35.73 ± 9.76 ^a^	0.609 ^d^
**BMI (kg/m^2^)**	26.86 ± 2.38 ^a^	26.21 ± 2.19 ^a^	26.85 ± 2.39 ^a^	0.253 ^d^
**EDSS**	2.50 (2.50–3) ^b^	2.50 (2–3) ^b^	2.50 (2–3) ^b^	0.419 ^e^
**Glucose (mg/dL)**	94 (84–104) ^b^	89 (84–96) ^b^	88.50 (83.75–94) ^b^	0.206 ^e^
**Creatinine (mg/dL)**	0.79 (0.73–0.98) ^b,k^	0.73 (0.64–0.85) ^b^	0.69 (0.64–0.76) ^b,k^	*p* = 0.013 ^e,^* p_k_ = 0.005
**ALT (U/L)**	27 (11–53) ^b^	26.35 (14.32–49) ^b^	26.50 (18–42.25) ^b^	0.931 ^e^
**AST (U/L)**	27 (13–32) ^b^	20.50 (16–30.75) ^b^	23 (17–26) ^b^	0.962 ^e^
**GGT (U/L)**	37 (26–58) ^b,k,l^	20.50 (13–41.75) ^b,k^	21 (13.50–30.75) ^b,l^	*p* = 0.032 ^e,^* p_k_ = 0.033 *p*_l_ = 0.005
**LDL cholesterol (mg/dL)**	128 (112.75–139.82) ^b^	117.50 (90.75–139.50) ^b^	107 (79–149) ^b^	0.238 ^e^
**HDL cholesterol (mg/dL)**	49.40 (39.50–60.75) ^b,k^	56 (48.20–66) ^b^	62 (52–72.97) ^b,k^	*p* = 0.027 ^e,^* p_k_ = 0.013
**Total cholesterol (mg/dL)**	199.75 ± 61.45 ^a^	198.12 ± 57.90 ^a^	195.63 ± 47.69 ^a^	0.965 ^d^
**Triglyceride (mg/dL)**	175 (81.15–241.25) ^b^	85 (68.50–129.50) ^b^	86 (59.25–151.75) ^b^	0.072 ^e^
**Leukocyte (** **×** **10^3^/µL)**	6.83 ± 2.19 ^a,k,l^	4.88 ± 1.34 ^a,k^	4.22 ± 1.63 ^a,l^	*p* < 0.001 ^d,^* p_k_ < 0.001 *p*_l_ < 0.001
**Neutrophil (** **×** **10^3^/µL)**	4.54 (3.56–5.63) ^b,k,l^	3.55 (2.64–4.28) ^b,k^	3.17 (2.51–3.89) ^b,l^	*p* = 0.003 ^e,^* p_k_ = 0.007 *p*_l_ = 0.002
**Lymphocyte (** **×** **10^3^/µL)**	1.31 (1.16–1.78) ^b,k,l^	0.64 (0.56–0.76) ^b,k,m^	0.36 (0.29–0.43) ^b,l,m^	*p* < 0.001 ^e,^* p_k_ < 0.001 *p*_l_ < 0.001 p_m_ < 0.001
**Monocyte (** **×** **10^3^/µL)**	0.48 ± 0.14 ^a^	0.46 ± 0.12 ^a^	0.42 ± 0.14 ^a^	0.141 ^d^
**Hemoglobin (g/dL)**	14.07 ± 1.82 ^a,k^	13.78 ± 1.62 ^a,l^	12.97 ± 1.54 ^a,k,l^	*p* = 0.015 ^d,^* p_k_ = 0.007 *p*_l_ = 0.035
**Hematocrit (%)**	41.61 ± 4.89 ^a,k^	41.14 ± 4.20 ^a,l^	38.70 ± 4.03 ^a,k,l^	*p* = 0.007 ^d,^* p_k_ = 0.037 *p*_l_ = 0.012
**Platelets (** **×** **10^3^/µL)**	280.10 ± 61.43 ^a^	258.95 ± 56.85 ^a^	243.56 ± 57.91 ^a^	0.069 ^d^
**MPV (fL)**	9.37 ± 1.22 ^a^	9.63 ± 1.48 ^a^	9.60 ± 1.07 ^a^	0.749 ^d^
**PCT (%)**	0.25 (0.22–0.28) ^b^	0.25 (0.20–0.27) ^b^	0.22 (0.20–0.25) ^b^	0.102 ^e^
**CRP (mg/dL)**	0.89 (0.20–2.85) ^b^	0.90 (0.53–2) ^b^	0.70 (0.44–1.70) ^b^	0.876 ^e^
**ESR (mm/h)**	10.50 (8.50–24.50) ^b^	7 (5–14) ^b^	11.50 (6–19.75) ^b^	0.077 ^e^
**TSH (mIU/L)**	1.72 ± 0.50 ^a^	1.73 ± 0.85 ^a^	1.65 ± 0.74 ^a^	0.888 ^d^
**Infection outcome**				
**Any infection**	0 (0%)	7 (9.3%)	5 (10.2%)	0.530 ^f^
**Infection type**				
**Acute gastroenteritis**	0 (0%)	2 (2.7%)	1 (2%)	
**Opportunistic infection**	0 (0%)	1 (1.3%)	2 (4.1%)	
**Acute nasopharyngitis**	0 (0%)	4 (5.3%)	2 (4.1%)	

^a^: Mean ± Standard deviation (SD); ^b^: Median (IQR: Interquartile Range); ^c^: Chi-Square Test; ^d^: ANOVA Test (post hoc: Bonferroni Test); ^e^: Kruskal–Wallis Test (Post hoc: Mann–Whitney U Test); ^f^: Fisher–Freeman–Halton Exact Test. *: Statistically significant (*p* < 0.05). Superscript letters ^k^, ^l^, and ^m^ indicate statistically significant pairwise differences within the corresponding row; groups sharing the same superscript letter differ significantly from each other. The corresponding pairwise *p* values are presented as p_k_, p_l_, and p_m_ in the *p*-value column.

**Table 6:** Exploratory logistic regression analysis of factors associated with Grade 4 lymphopenia at the 6-month follow-up.

Variable	Unadjusted OR (95% CI)	*p*	Adjusted OR (95% CI)	*p*
**Age**	0.991 (0.936–1.049)	0.742	0.989 (0.934–1.047)	0.693
**Female Sex**	2.564 (0.689–9.541)	0.160	2.883 (0.711–11.687)	0.138
**BMI**	0.907 (0.704–1.170)	0.453	-	-
**Baseline lymphocyte count**	0.084 (0.021–0.335)	<0.001 *	0.078 (0.018–0.325)	<0.001 *
**Baseline creatinine**	0.008 (0.001–1.048)	0.052	-	-

*: Statistically significant (*p* < 0.05). The dependent variable was Grade 4 lymphopenia at the 6-month follow-up. Patients without lymphopenia or with Grade 1–3 lymphopenia constituted the reference outcome category, and male sex was the reference category for sex. Age, sex, and baseline lymphocyte count were included in the multivariable model. Because only 15 Grade 4 events occurred, the analysis was considered exploratory. The multivariable model was statistically significant (χ^2^ = 20.493, df = 3, *p* < 0.001) and had a Nagelkerke R^2^ of 0.273. The Hosmer–Lemeshow test showed no evidence of poor model fit (χ^2^ = 5.520, df = 8, *p* = 0.701). No relevant multicollinearity was detected among the predictors (tolerance range, 0.978–0.998; VIF range, 1.002–1.023). Overall classification accuracy was 90.2%, with a sensitivity of 20.0% and a specificity of 98.4%. CI: Confidence interval; OR: Odds Ratio.

## Data Availability

The datasets generated and/or analyzed during the current study are available from the corresponding author on reasonable request.

## Abbreviations

ALC	Absolute lymphocyte count
ALT	Alanine aminotransferase
AST	Aspartate aminotransferase
BMI	Body mass index
BSA	Body surface area
CNS	Central nervous system
CrCl	Creatinine clearance
CTCAE	Common Terminology Criteria for Adverse Events
EDSS	Expanded Disability Status Scale
GGT	Gamma-glutamyl transferase
HSV	Herpes simplex virus
IgG	Immunoglobulin G
IgM	Immunoglobulin M
MS	Multiple sclerosis
NLR	Neutrophil-to-lymphocyte ratio
RRMS	Relapsing-remitting multiple sclerosis
SII	Systemic immune-inflammation index
VZV	Varicella-zoster virus

## References

[1] Jeffery D.R., Rammohan K.W., Hawker K., Fox E. (2016). Fingolimod: A review of its mode of action in the context of its efficacy and safety profile in relapsing forms of multiple sclerosis. Exp. Rev. Neurother..

[2] Pelletier D., Hafler D.A. (2012). Fingolimod for multiple sclerosis. N. Engl. J. Med..

[3] Saida T., Kikuchi S., Itoyama Y., Hao Q., Kurosawa T., Nagato K., Tang D., Zhang-Auberson L., Kira J. (2012). A randomized, controlled trial of fingolimod (FTY720) in Japanese patients with multiple sclerosis. Mult. Scler. J..

[4] Coyle P.K., Freedman M.S., Cohen B.A., Cree B.A.C., Markowitz C.E. (2024). Sphingosine 1-phosphate receptor modulators in multiple sclerosis treatment: A practical review. Ann. Clin. Transl. Neurol..

[5] Brinkmann V., Billich A., Baumruker T., Heining P., Schmouder R., Francis G., Aradhye S., Burtin P. (2010). Fingolimod (FTY720): Discovery and Development of an Oral Drug to Treat Multiple Sclerosis. Nat. Rev. Drug Discov..

[6] Samadzade U., Alizada S., Çalışkan C., Mammadov O., Ozakbas S. (2026). No evidence of disease activity after fingolimod treatment: A three-year real-world comparison of pre- and post-treatment outcomes in multiple sclerosis. Mult. Scler. Relat. Disord..

[7] Cohen J.A., Barkhof F., Comi G., Hartung H.P., Khatri B.O., Montalban X., Pelletier J., Capra R., Gallo P., Izquierdo G. (2010). Oral fingolimod or intramuscular interferon for relapsing multiple sclerosis. N. Engl. J. Med..

[8] Kappos L., Radue E.W., O’Connor P., Polman C., Hohlfeld R., Calabresi P., Selmaj K., Agoropoulou C., Leyk M., Zhang-Auberson L. (2010). A placebo-controlled trial of oral fingolimod in relapsing multiple sclerosis. N. Engl. J. Med..

[9] Novartis Pharmaceuticals UK Ltd Gilenya 0.5 mg Hard Capsules. Electronic Medicines Compendium (eMC). Updated 30 June 2017. https://www.medicines.org.uk/emc/medicine/24443.

[10] Teniente-Serra A., Hervás J.V., Quirant-Sánchez B., Mansilla M.J., Grau-López L., Ramo-Tello C., Martínez-Cáceres E.M. (2016). Baseline differences in minor lymphocyte subpopulations may predict response to fingolimod in relapsing-remitting multiple sclerosis patients. CNS Neurosci. Ther..

[11] Polman C.H., Reingold S.C., Banwell B., Clanet M., Cohen J.A., Filippi M., Fujihara K., Havrdova E., Hutchinson M., Kappos L. (2011). Diagnostic criteria for multiple sclerosis: 2010 revisions to the McDonald criteria. Ann. Neurol..

[12] Balzano N., Di Napoli R., Fraenza F., Cesare D.D.G., Moreggia O., Cardillo M., Scavone C., Maniscalco G.T., Capuano A., Sportiello L. (2025). Lymphopenia associated with sphingosine 1-phosphate receptor modulators (S1PRMs) in multiple sclerosis: Analysis of European pharmacovigilance data. Pharmacol. Rep..

[13] Nikitina V., Laurini G.S., Montanaro N., Motola D. (2024). Safety of fingolimod in patients with relapsing-remitting multiple sclerosis: A descriptive analysis of data from the EudraVigilance database. J. Neurol. Sci..

[14] Huh S.-Y., Kim S.-H., Kim K.H., Kwon Y.N., Kim S.-M., Kim S.W., Shin H.Y., Chung Y.H., Min J.-H., So J. (2022). Safety and temporal pattern of the lymphocyte count during fingolimod therapy in patients with multiple sclerosis: Real-world Korean experience. J. Clin. Neurol..

[15] Zhao Z., Ma C.-L., Gu Z.-C., Dong Y., Lv Y., Zhong M.-K. (2021). Incidence and risk of infection associated with fingolimod in patients with multiple sclerosis: A systematic review and meta-analysis of 8,448 patients from 12 randomized controlled trials. Front. Immunol..

[16] Sarıkaya C., Gacar G., Efendi H. (2024). Effect of Fingolimod on lymphocyte subsets in patients with relapsing multiple sclerosis. Cureus.

[17] Dominguez Mozo M.I., Galán V., Ramió Torrentà L., Quiroga A., Quintana E., Villar L.M., Costa-Frossard L., Fernández-Velasco J.I., Villarrubia N., Garcia-Martinez M.A. (2024). A two-year real-world study with fingolimod: Early predictors of efficacy and an association between EBNA-1 IgG titers and multiple sclerosis progression. Front. Immunol..

[18] Sato N., Wakimoto K., Kato K., Susuta Y., Ueda K., Satou Y., Sasajima T., Kira J.I. (2024). Safety and effectiveness of fingolimod in Japanese patients with multiple sclerosis: Results of a post-marketing surveillance study. Clin. Exp. Neuroimmunol..

